# Prognostic role of lymph node regression in patients with esophageal cancer undergoing neoadjuvant therapy

**DOI:** 10.3389/pore.2024.1611844

**Published:** 2024-10-11

**Authors:** Pingrun Chen, Maojia Chen, Yijie Bu, Guowei Che, Chao Cheng, Yan Wang

**Affiliations:** ^1^ Department of Gastroenterology and Hepatology, West China Hospital, Sichuan University, Chengdu, China; ^2^ Animal Experiment Center, West China Hospital, Sichuan University, Chengdu, China; ^3^ Department of Thoracic Surgery, West China Hospital, Sichuan University, Chengdu, China; ^4^ Department of Thoracic Surgery/Lung Cancer Center, West China Hospital, Sichuan University, Chengdu, China

**Keywords:** lymph node regression, esophageal cancer, neoadjuvant therapy, survival, meta-analysis

## Abstract

**Purpose:**

To clarify the prognostic value of lymph node regression (LNR) status including the lymph node regression grade (LNRG) and N downstaging in patients with esophageal cancer receiving neoadjuvant therapy based on available evidence.

**Methods:**

Several databases were searched up to 25 March 2024. The main outcomes included overall survival (OS), disease-free survival (DFS) and cancer-specific survival (CSS). Hazard ratios (HRs) and 95% confidence intervals (CIs) were combined. Subgroup analyses based on the neoadjuvant therapy and pathological type were also conducted.

**Results:**

In total, 14 retrospective studies with 3,212 participants were included. Nine and five studies explored the relationship between LNRG and N downstaging and survival, respectively. Pooled results indicated that complete LNR predicted significantly improved OS (HR = 0.47, 95% CI: 0.41–0.55, P < 0.001) and DFS (HR = 0.42, 95% CI: 0.32–0.55, P < 0.001) and subgroup analysis based on neoadjuvant therapy and pathological type manifested similar results. Besides, N downstaging was also significantly related to improved OS (HR = 0.40, 95% CI: 0.21–0.77, P = 0.006) and CSS (HR = 0.27, 95% CI: 0.12–0.60, P < 0.001).

**Conclusion:**

LNR could serve as a novel and reliable prognostic factor in patients with esophageal cancer receiving neoadjuvant therapy and complete LNR and N downstaging predict better survival.

## Introduction

Esophageal cancer ranks as the fourth leading cause of cancer-related deaths, comprising predominantly squamous cell carcinoma and adenocarcinoma, with an overall poor prognosis [1, 2]. In recent years, the clinical role of neoadjuvant therapy in the treatment of esophageal cancer has become increasingly prominent and neoadjuvant therapy combined with surgery significantly improves the prognosis for the majority of patients with locally advanced esophageal cancer [3]. Recent studies have demonstrated the superiority of neoadjuvant chemoradiotherapy over neoadjuvant chemotherapy in terms of long-term survival outcomes in locally advanced esophageal cancer [4]. Even with similar adverse effects, neoadjuvant chemoradiotherapy provides superior short-term benefits compared to neoadjuvant chemotherapy [4]. Moreover, neoadjuvant chemoradiotherapy is associated with impressive rates of pathological complete response, and survival [5, 6]. In addition, the role of neoadjuvant immunotherapy is gradually being elucidated in the clinic [7]. Thus, neoadjuvant therapy holds an indispensable position in the comprehensive antitumor therapy of esophageal cancer.

Furthermore, with the incorporation of adjuvant therapy, the TNM stage appears to be less reliable in predicting the prognosis of neoadjuvant therapy compared to surgery alone. Therefore, to aid in prognosis evaluation, imaging and pathological approaches to assess tumor regression are considered vital evaluation tools. However, pathological criteria undoubtedly offer greater objectivity. The widely adopted tumor regression grade (TRG) system categorizes residual tumors after treatment-induced regression based on parameters such as quantity, proportion, tumor cell condition, and extent and distribution of fibrosis [8]. Nevertheless, there exist multiple variations of TRG [9, 10]. While the majority of evaluation systems are generally regarded as good indicators for predicting short-term therapeutic effects and estimating the risk of recurrence, each grade only reflects the therapeutic effect on the primary tumors [9, 10]. However, it has been reported that the response of primary lesions and lymph nodes to neoadjuvant therapy differs [9]. Furthermore, some studies have indicated that lymph node metastasis status is more strongly associated with prognosis than primary lesion invasion, whether after surgery or neoadjuvant therapy [11]. Consequently, lymph nodes must be individually evaluated and the prognostic role of lymph node regression (LNR) in patients with esophageal cancer should be further determined. For LNR grade (LNRG) assessment, dissected lymph nodes are usually stained with hematoxylin and eosin and analyzed microscopically for metastatic disease in clinics. Currently, there is no standardized protocol for evaluating LNR. Lymph node downstaging is usually defined as any regional lymph node that is positive on clinical evaluation (cN+) and subsequently has no evidence of pathologic regional lymph node disease. To date, the prognostic role of LNR in esophageal cancer patients undergoing neoadjuvant therapy remains uncertain.

Therefore, this study aimed to identify the predictive role of LNR status in locally advanced patients with esophageal cancer who received neoadjuvant therapy based on current evidence.

## Materials and methods

This study was performed according to the Preferred Reporting Items for Systematic Review and Meta-Analyses 2020 [12].

### Literature search

The Medline, EMBASE, and Web of Science databases were searched from their inception up to 25 March 2024. The following terms were used: esophageal, esophagus, tumor, cancer, neoplasm, carcinoma, survival, prognostic, prognosis, neoadjuvant, lymph node regression, and N downstaging. A detailed search strategy in Medline is provided in Supplementary file 1. References to the included studies were also reviewed.

### Inclusion criteria

Studies were included if they met the following criteria: 1) patients were pathologically diagnosed with primary esophageal cancer; 2) patients received neoadjuvant therapy including chemotherapy, radiotherapy, immunotherapy, or combined therapy; 3) lymph node status was evaluated before and after neoadjuvant therapy; 4) patients in the complete/subtotal response group and partial/no response group and patients in the N downstaging group (ypN0) and ypN+ group were compared separately; 5) overall survival (OS), disease-free survival (DFS) or (and) was compared between groups; 6) hazard ratios (HRs) with 95% confidence intervals (CIs) were reported, or the Kaplan-Meier survival curves were provided; 7) full texts were available in English.

### Exclusion criteria

Studies were excluded if they met the following criteria: 1) insufficient, duplicated, or overlapping data; 2) animal studies, editorials, letters, meeting abstracts or case reports.

### Data extraction

The following information was collected: the first author, publication year, country, sample size, c-tumor-node-metastasis (cTNM) stage, type of neoadjuvant therapy, pathological type, type of LNR, definition of LNRG or N downstaging, source of HR, follow-up time, outcome, and HR with 95% CI.

### Quality evaluation

The Newcastle-Ottawa scale (NOS) was used to evaluate the quality of included retrospective studies and studies with an NOS score ≥6 were regarded as high-quality studies [13].

The literature search, selection, data extraction, and quality evaluation were all performed by two authors independently and all disagreements were resolved by team discussion.

### Statistical analysis

All statistical analyses were conducted using STATA 15.0 software. HR with 95% CI was combined to identify the association between LNR and survival in patients with esophageal cancer undergoing neoadjuvant therapy. Heterogeneity among included studies was evaluated by I^2^ statistics and Q tests. When significant heterogeneity was observed, presenting as I^2^ > 50% or P < 0.1, the random-effects model was applied; otherwise, the fixed-effects model was used [14]. Subgroup analysis focusing on neoadjuvant therapy and pathological type was also conducted. Sensitivity analysis was conducted to evaluate the stability of the pooled results. Begg’s funnel plot and Egger’s test were conducted to detect publication bias [15, 16]. Significant publication bias was defined as P < 0.05.

## Results

### Literature search and selection

**FIGURE 1:**
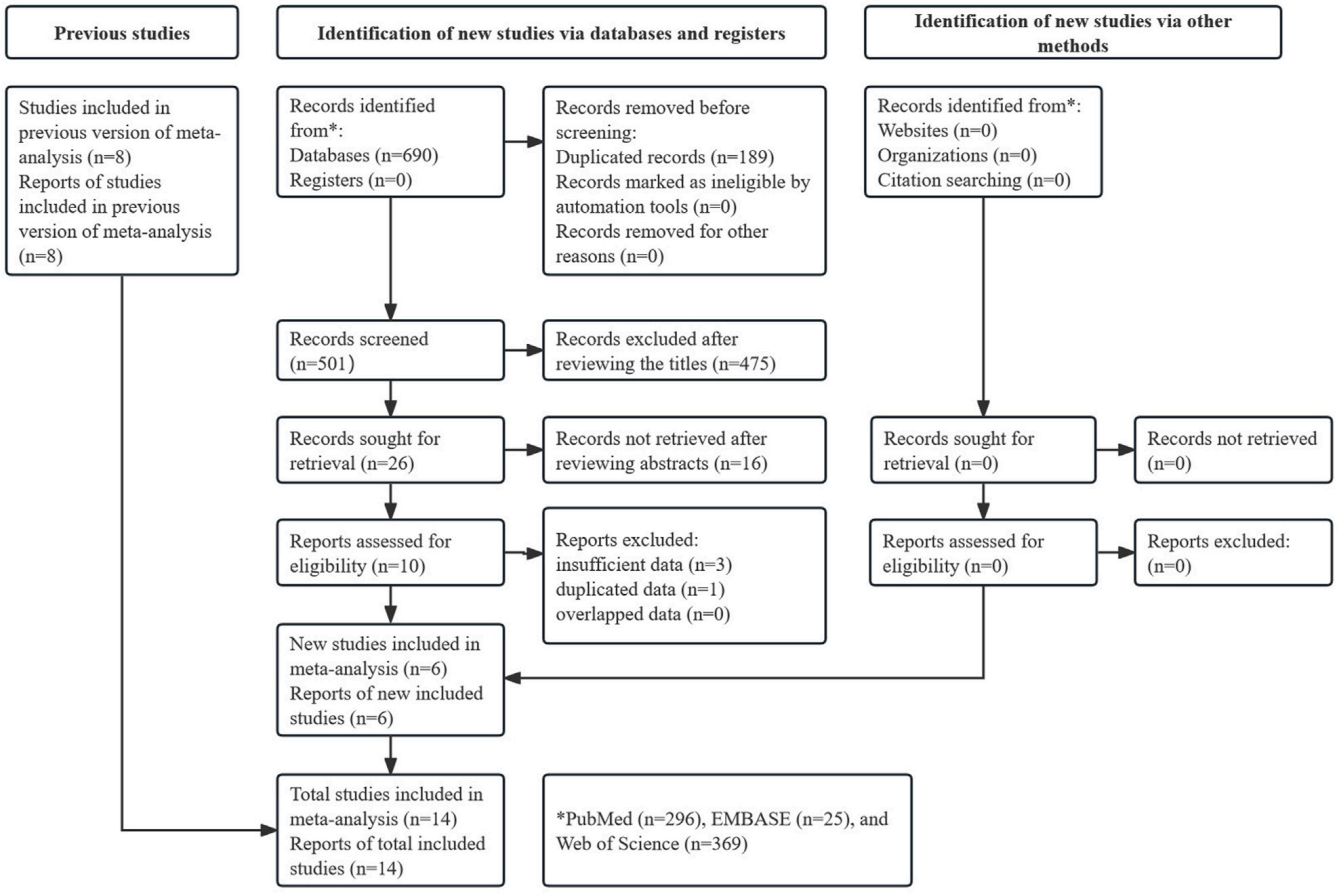
Prisma flow diagram of this meta-analysis.

As shown in Figure 1, 690 records were identified from three databases and 189 duplicate records were removed. After reviewing the titles and abstracts, 491 records were excluded. Finally, 14 studies were included after reviewing the full text of the remaining publications [17–30]. Notably, eight studies were included in a previous similar meta-analysis by Hagens et al. [31]. Therefore, six new studies were included in this updated meta-analysis.

### Baseline characteristics of included studies

Among the 14 included retrospective studies, 3,212 patients were enrolled. Nine studies explored the relationship between LNRG and prognosis [18, 20, 21, 25–30], while the other five studies identified the predictive role of N downstaging for survival [17, 19, 22–24]. The sample sizes ranged from 40 to 981 subjects. The majority of patients received chemoradiotherapy as neoadjuvant therapy. Notably, neoadjuvant therapy was not incorporated in all included studies. All included studies directly reported HRs with 95% CIs and were high-quality studies with the NOS score ≥6. Further detailed information is presented in Table 1.

**TABLE 1:** Basic characteristics of the included studies.

Author	Year	Country	Sample size	cTNM stage	Neoadjuvant therapy	Pathological type	LNR type	Definition of LNRG/N downstaging	Source of HR	Follow-up period	Endpoint	NOS score
Bollschweiler [18]	2011	Germany	40	cT3/4N+	CRT	Mixed	LNRG	Low-risk: no LNM and less than three LNs with central fibrosis; medium-risk: no LNM and three or more LNs with central fibrosis *or* LNM with an LN ratio less than 0.05; high-risk: LNM	R	5.5 (4.2–7.6) years (median)	OS	6
Nieman [20]	2015	USA	90	cIB-cIIIA	CRT	AC	LNRG	Complete response: without evidence of metastatic disease; partial response: with evidence of previous cancer involvement but no currently viable cancer cells; no response: involved with malignancy	R	27 (13.4–40.1) months (median)	OS	7
Philippron [21]	2016	Germany	222	cT3N + M0	CRT	Mixed	LNRG	High-LN response: without LNM showing central fibrosis in fewer than three LNs; medium-LN response: central fibrosis in three or more LNs of ypN0 or LNM with an LN ratio of less than 0.05; Low-LN response: other	R	4.5 (1.0–11.2) years (median)	OS	7
Davies [25]	2018	Sweden	183	cT2-4N+	CT	AC	LNRG	Score 1: complete response; Score 2: <10% residual tumor; Score 3: 10%–50% residual tumor; Score 4: >50% viable tumor; Score 5: no response	R	NR	OS, DFS	7
Hsu [26]	2021	China	115	cT1-4N−/+	CRT	SCC	LNRG	Score 0: N (−) with no evidence of tumor involvement or regression; score 1: N (−) with evidence of complete regression; score 2: N (+) with\50% viable tumor; score 3: N (+) with >50% viable tumor	R	NR	OS, DFS	7
Koemans [27]	2021	Netherlands	117	cIB–IIIC	CRT	Mixed	LNRG	Class A: no tumor, no signs of regression; B: tumor without regression; C: viable tumor and regression; D: complete response	R	37 (29–44) months (median)	OS	7
Evans [28]	2022	Italy	130	cT2-4N+	CT	AC	LNRG	Complete response: any of the following features with no residual tumor cells: the presence of foamy or hemosiderin-laden macrophages with or without dystrophic calcification, (ii) lakes of acellular mucin, and/or (iii) the presence of substantial fibrosis or tumor necrosis; partial response: with features of nodal regression and persistent tumor cells; no response: with LN metastasis but no features of regression	R	NR	OS, DFS	7
Moore [29]	2023	UK	763	cT1-4N−/+	CT	AC	LNRG	Score 1: complete response; score 2: <10% residual tumor; score 3: 10%–50% residual tumor; score 4: >50% residual tumor; score 5: no response	R	NR	OS, DFS	8
Yehan [30]	2024	China	112	cN+	CRT	SCC	LNRG	Score 1: 0% of total viable tumor area; score 2: <10% of total viable tumor area; score 3: 10%–50% of total viable tumor area; score 4: >50% of total viable tumor area	R	29.6 months (median)	DFS	7
Rice [17]	2001	USA	69	cN+	NR	Mixed	N downstaging	cN1 to ypN0	R	26 ± 15 months (mean)	OS	6
Donohoe [19]	2013	Ireland	155	cN+	CRT	Mixed	N downstaging	cN+ to ypN0	R	48 (6–273) months (median)	OS	6
Zanoni [22]	2016	Italy	55	cN+	CRT	Mixed	N downstaging	cN+ to ypN0	R	44 (11–131) months (median)	OS, CSS	6
Noble [23]	2017	UK	981	NR	CRT	AC	N downstaging	cN+ to ypN0	R	NR	OS	6
Shapiro [24]	2017	Netherland	180	cN+	CRT	Mixed	N downstaging	cN+ to ypN0	R	NR	OS	6

TNM: tumor-node-metastasis; CRT: chemoradiotherapy; CT: chemotherapy; LNR: lymph node regression; LNRG: lymph node regression grade; LNM: lymph node metastasis; LN: lymph node; AC: adenocarcinoma; SCC: squamous cell carcinoma; R: reported; OS: overall survival; DFS: disease-free survival; CSS: cancer-specific survival; NOS: Newcastle-Ottawa Scale.

### Association of LNRG with survival in patients with esophageal cancer receiving neoadjuvant therapy

**FIGURE 2:**
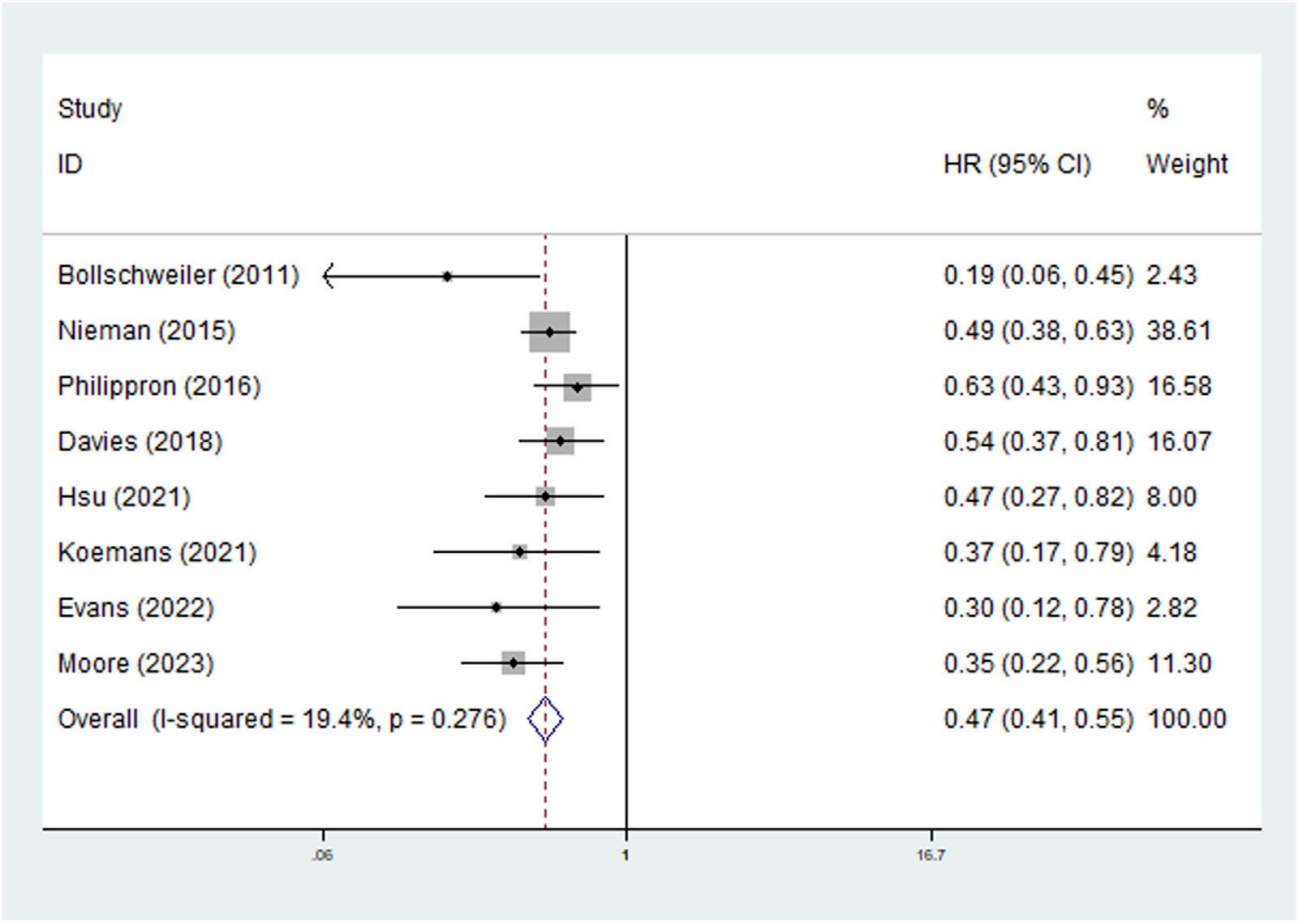
The association between lymph node regression grade and overall survival in patients with esophageal cancer undergoing neoadjuvant therapy.

The survival of patients with complete (or subtotal) lymph node response and with partial (or no) lymph node response was compared in the included studies. Eight studies explored the predictive role of LNRG for OS, and the pooled results demonstrated that complete LNR predicted significantly improved OS (HR = 0.47, 95% CI: 0.41–0.55, P < 0.001; I^2^ = 19.4%, P = 0.276) (Figure 2). Then, subgroup analysis stratified by neoadjuvant therapy (chemoradiotherapy: HR = 0.49, 95% CI: 0.41–0.59, P < 0.001; chemotherapy: HR = 0.43, 95% CI: 0.33–0.58, P < 0.001) and pathological type (adenocarcinoma: HR = 0.46, 95% CI: 0.38–0.56, P < 0.001; squamous cell carcinoma: HR = 0.47, 95% CI: 0.27–0.82, P = 0.008) showed similar results (Table 2).

**TABLE 2:** Results of the meta-analysis.

	No. of studies	HR	95% CI	p-Value	I^2^ (%)	P_heterogeneity_
LNRG						
Overall survival	8	0.47	0.41–0.55	<0.001	19.4	0.276
Type of neoadjuvant therapy						
Chemoradiotherapy	5	0.49	0.41–0.59	<0.001	28.1	0.234
Chemotherapy	3	0.43	0.33–0.58	<0.001	23.3	0.271
Pathological type						
Adenocarcinoma	4	0.46	0.38–0.56	<0.001	0.0	0.394
Squamous cell carcinoma	1	0.47	0.27–0.82	0.008	-	-
Disease-free survival	5	0.42	0.32–0.55	<0.001	30.0	0.221
Type of neoadjuvant therapy						
Chemoradiotherapy	2	0.53	0.33–0.87	0.011	70.9	0.064
Chemotherapy	3	0.38	0.28–0.52	<0.001	0.0	0.605
Pathological type						
Adenocarcinoma	2	0.53	0.33–0.87	0.011	70.9	0.064
Squamous cell carcinoma	3	0.38	0.28–0.52	<0.001	0.0	0.605
N downstaging						
Overall survival	5	0.40	0.21–0.77	0.006	96.6	<0.001
Cancer-specific survival	1	0.27	0.12–0.60	<0.001	-	-

HR, hazard ratio; CI, confidence interval.

**FIGURE 3:**
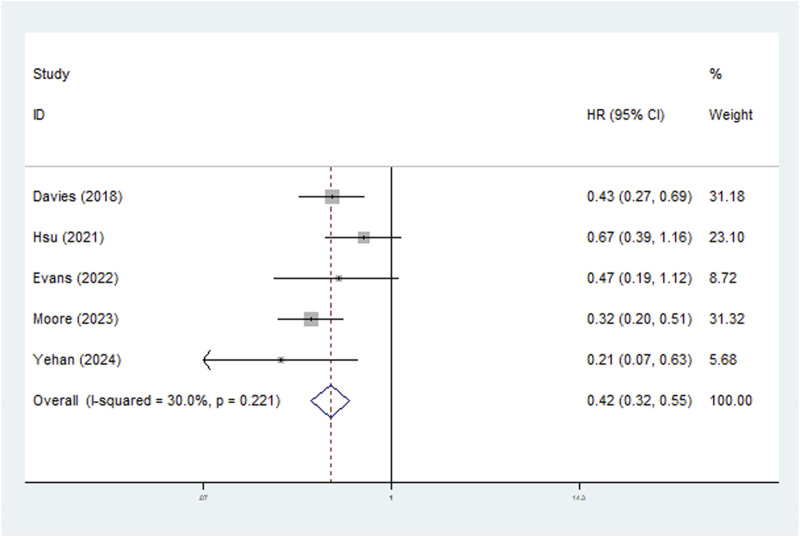
The association between lymph node regression grade and disease-free survival in patients with esophageal cancer undergoing neoadjuvant therapy.

Five studies explored the relationship between LNRG and DFS and the pooled results indicated that LNRG was significantly associated with DFS (HR = 0.42, 95% CI: 0.32–0.55, P < 0.001; I^2^ = 30.0%, P = 0.221) (Figure 3). Similarly, subgroups based on neoadjuvant therapy (chemoradiotherapy: HR = 0.53, 95% CI: 0.33–0.87, P = 0.011; chemotherapy: HR = 0.38, 95% CI: 0.28–0.52, P < 0.001) and pathological type (adenocarcinoma: HR = 0.53, 95% CI: 0.33–0.87, P = 0.011; squamous cell carcinoma: HR = 0.38, 95% CI: 0.28–0.52, P < 0.001) identified the association of complete LNR with improved DFS (Table 2).

### Association of N downstaging with survival in patients with esophageal cancer receiving neoadjuvant therapy

**FIGURE 4:**
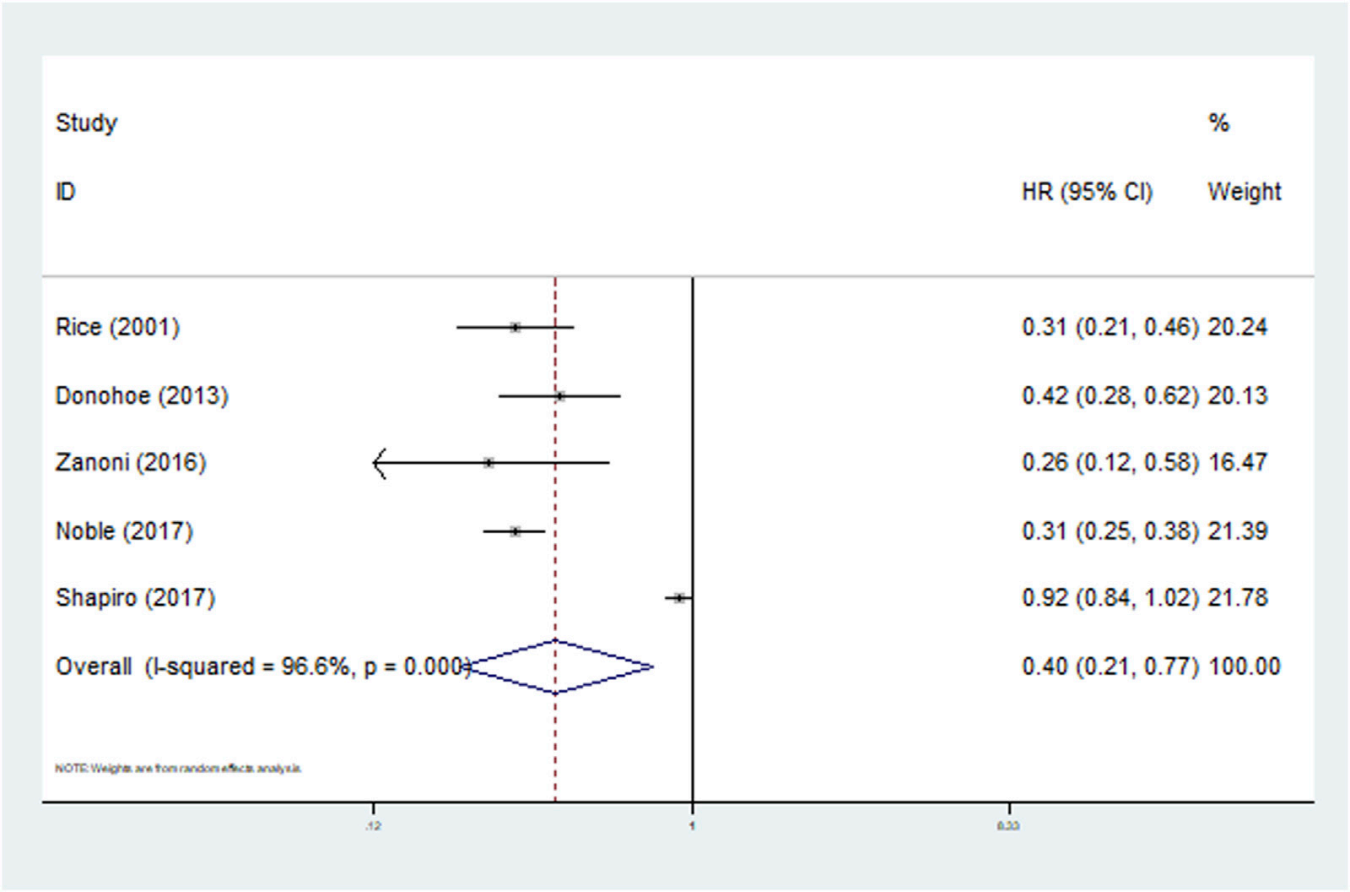
The association between N downstaging and overall survival in patients with esophageal cancer undergoing neoadjuvant therapy.

All included studies defined the N downstaging as cN+ to ypN0. Five studies clarified the predictive role of N downstaging for OS and the pooled results demonstrated that N downstaging predicted significantly worse OS (HR = 0.40, 95% CI: 0.21–0.77, p = 0.006; I^2^ = 96.6%, P < 0.001) (Figure 4). However, due to the limited data available, it was not possible to conduct a more detailed subgroup analysis on N downstaging for OS.

Furthermore, Zanoni et al. reported that N downstaging was also significantly associated with improved CSS (HR = 0.27, 95% CI: 0.12–0.60, P < 0.001) (Table 2).

### Sensitivity analysis and publication bias

**FIGURE 5:**
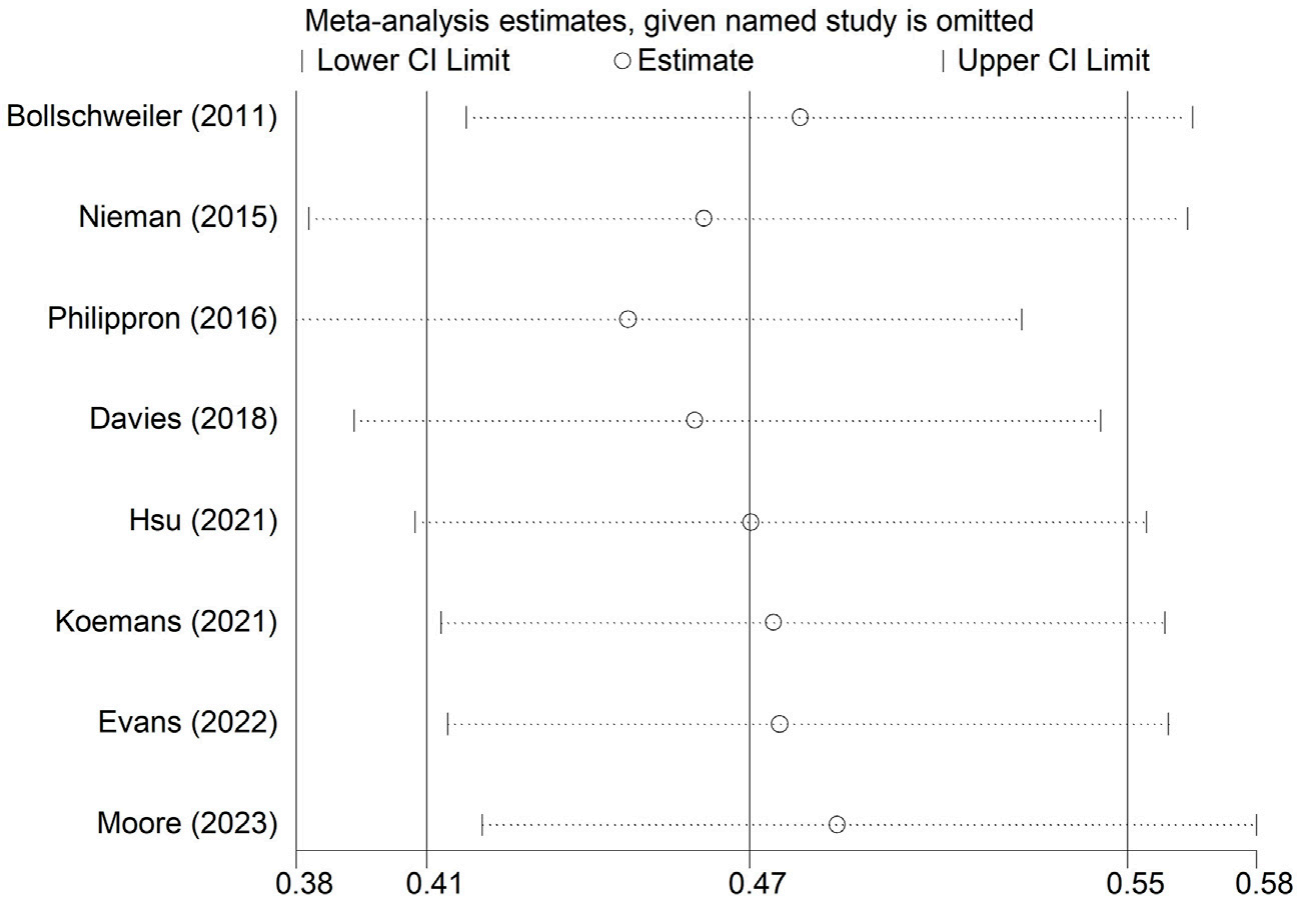
Sensitivity analysis for the association between lymph node regression grade and overall survival in patients with esophageal cancer undergoing neoadjuvant therapy.

We conducted a sensitivity analysis on LNRG for OS. As shown in Figure 5, the pooled results of this meta-analysis were stable and reliable.

**FIGURE 6:**
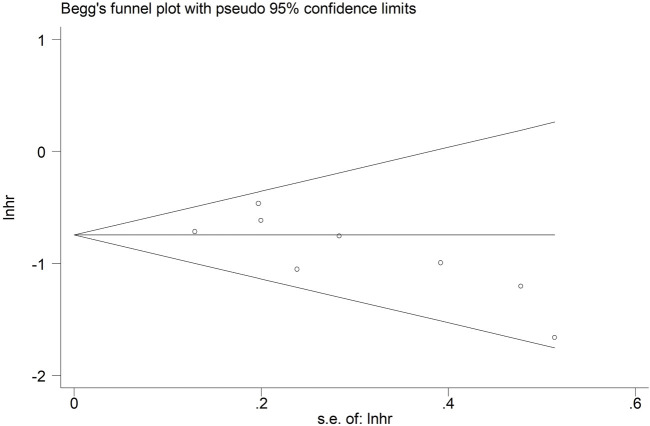
Begg’s funnel plot for the association between lymph node regression grade and overall survival in patients with esophageal cancer undergoing neoadjuvant therapy.

Meanwhile, Begg’s funnel plot (Figure 6) and Egger’s test (P = 0.064) indicated that there was no obvious publication bias.

## Discussion

According to this meta-analysis, LNR is significantly associated with long-term survival among patients with esophageal cancer who receive neoadjuvant therapy and patients with complete (or subtotal) LNR or (and) N downstaging are more likely to experience a better prognosis. Therefore, LNR should be carefully considered as a prognostic indicator in this group of patients.

Hagens et al. conducted a similar meta-analysis to explore the prognostic role of LNR in esophageal cancer [31]. They included eight studies, three of which assessed LNRG and five of which assessed N downstaging. According to their results, N downstaging was associated with improved survival (HR = 0.41, 95% CI: 0.22–0.77, P = 0.005). However, no significant association was observed between LNRG and survival (HR = 0.52, 95% CI: 0.26–1.06). Furthermore, a more detailed analysis was not performed in their meta-analysis [31]. Therefore, we conducted the current meta-analysis to further identify the prognostic relevance of LNR on survival in patients with esophageal cancer undergoing neoadjuvant therapy and to provide more evidence on the prognostic role of LNR in this group of patients.

The response to chemoradiotherapy may differ between lymph node metastases and primary lesions in esophageal cancer. First, cancer cells in primary lesions and metastases may exhibit different levels of differentiation [32]. Typically, cancer cells in primary lesions may be more differentiated, while those in metastases may be more unstable, heterogeneous, and may even contain more stem cell-like cells [33, 34]. This could lead to an increased resistance of metastases to chemotherapy [34]. Second, there may be differences in the microenvironment between metastatic lymph nodes and primary tumors, such as oxygen concentration, nutrient supply, and immune cell infiltration [35, 36]. These microenvironmental differences may affect the sensitivity of cancer cells to chemoradiotherapy [37]. Additionally, metastases may accumulate genetic mutations different from those in primary lesions, which may affect the sensitivity of cancer cells to anti-tumor drugs [38]. Specifically, metastases may develop resistance mutations to certain drugs [38]. While previous studies have often assessed the efficacy of neoadjuvant therapy based on changes in primary tumors, the evaluation of LNR status is also crucial in practice.

However, there is currently no standardized protocol for evaluating LNR, leading to variations in the grading systems used in the included studies, which needs to be further elucidated in future studies. At the same time, we believe it is also crucial to compare and assess the role of regression in primary tumors and lymph node metastases in predicting the prognosis of patients with esophageal cancer undergoing neoadjuvant therapy or to investigate the prognostic significance of joint evaluation of regression in primary tumors and lymph node metastases in response to neoadjuvant therapy. As reported by Yun et al., the combined evaluation of TRG and lymph node status demonstrated greater clinical value in predicting the prognosis of patients with esophageal cancer undergoing neoadjuvant therapy compared to TNM stage, TRG, and LNR [9]. Furthermore, whether LNR status contributes to guiding the formulation of postoperative adjuvant therapy or treatment strategies after recurrence in patients with esophageal cancer also needs to be explored.

Our meta-analysis has several limitations. First, the sample size was relatively small and all included studies were retrospective. Second, there were some confounding factors such as the surgical procedures, definition of LNRG, tumor stage, etc. Third, regarding LNRG, some studies included a small number of cN- patients, but did not specify whether these patients were excluded during the analysis, which may introduce a slight bias. Fourth, data from other countries were missing, more studies in other countries are still needed to verify our findings. Fifth, the neoadjuvant immunotherapy was not considered in all included studies. The association of LNR with prognosis in patients with esophageal cancer receiving neoadjuvant immunotherapy should be further investigated. Sixth, the optimal neoadjuvant chemotherapy or chemoradiotherapy regimen remains controversial, especially in patients with different clinical characteristics.

## Conclusion

Overall, LNR could serve as a novel and reliable prognostic factor in patients with esophageal cancer undergoing neoadjuvant therapy and complete LNR and N downstaging predict better prognosis.

## Data Availability

The original contributions presented in the study are included in the article/Supplementary Material, further inquiries can be directed to the corresponding authors.
