# Supplementary figures and images for: Prognostic role of lymph node regression in patients with esophageal cancer undergoing neoadjuvant therapy
Source: Pathol Oncol Res. 2024 Oct 11;30:1611844. doi: 10.3389/pore.2024.1611844 (PMC11502349; doi:10.3389/pore.2024.1611844)

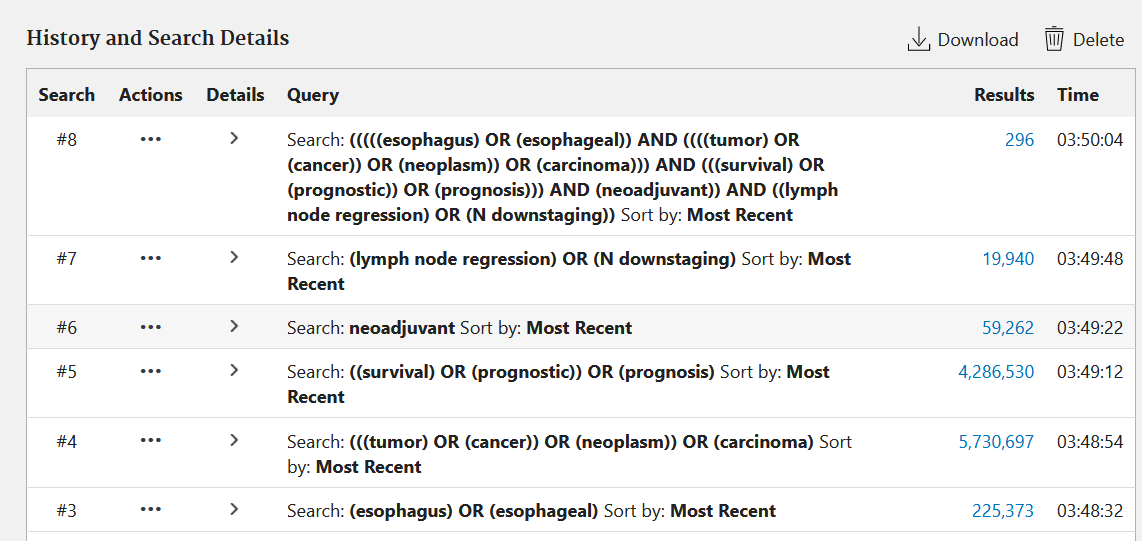

Supplement: Supplementary file 1 [file DataSheet1.DOCX]
